# Incorporating Domain Knowledge Into Language Models by Using Graph Convolutional Networks for Assessing Semantic Textual Similarity: Model Development and Performance Comparison

**DOI:** 10.2196/23101

**Published:** 2021-11-26

**Authors:** David Chang, Eric Lin, Cynthia Brandt, Richard Andrew Taylor

**Affiliations:** 1 Yale Center for Medical Informatics Yale University New Haven, CT United States; 2 Department of Psychiatry Yale University School of Medicine New Haven, CT United States; 3 Department of Emergency Medicine Yale University School of Medicine New Haven, CT United States; 4 West Haven Campus Veterans Affairs Connecticut Healthcare System West Haven, CT United States

**Keywords:** natural language processing, graph neural networks, National NLP Clinical Challenges, bidirectional encoder representation from transformers

## Abstract

**Background:**

Although electronic health record systems have facilitated clinical documentation in health care, they have also introduced new challenges, such as the proliferation of redundant information through the use of copy and paste commands or templates. One approach to trimming down bloated clinical documentation and improving clinical summarization is to identify highly similar text snippets with the goal of removing such text.

**Objective:**

We developed a natural language processing system for the task of assessing clinical semantic textual similarity. The system assigns scores to pairs of clinical text snippets based on their clinical semantic similarity.

**Methods:**

We leveraged recent advances in natural language processing and graph representation learning to create a model that combines linguistic and domain knowledge information from the MedSTS data set to assess clinical semantic textual similarity. We used bidirectional encoder representation from transformers (BERT)–based models as text encoders for the sentence pairs in the data set and graph convolutional networks (GCNs) as graph encoders for corresponding concept graphs that were constructed based on the sentences. We also explored techniques, including data augmentation, ensembling, and knowledge distillation, to improve the model’s performance, as measured by the Pearson correlation coefficient (*r*).

**Results:**

Fine-tuning the BERT_base and ClinicalBERT models on the MedSTS data set provided a strong baseline (Pearson correlation coefficients: 0.842 and 0.848, respectively) compared to those of the previous year’s submissions. Our data augmentation techniques yielded moderate gains in performance, and adding a GCN-based graph encoder to incorporate the concept graphs also boosted performance, especially when the node features were initialized with pretrained knowledge graph embeddings of the concepts (*r*=0.868). As expected, ensembling improved performance, and performing multisource ensembling by using different language model variants, conducting knowledge distillation with the multisource ensemble model, and taking a final ensemble of the distilled models further improved the system’s performance (Pearson correlation coefficients: 0.875, 0.878, and 0.882, respectively).

**Conclusions:**

This study presents a system for the MedSTS clinical semantic textual similarity benchmark task, which was created by combining BERT-based text encoders and GCN-based graph encoders in order to incorporate domain knowledge into the natural language processing pipeline. We also experimented with other techniques involving data augmentation, pretrained concept embeddings, ensembling, and knowledge distillation to further increase our system’s performance. Although the task and its benchmark data set are in the early stages of development, this study, as well as the results of the competition, demonstrates the potential of modern language model–based systems to detect redundant information in clinical notes.

## Introduction

Electronic health records (EHRs) have introduced efficiencies in clinical documentation via the automatic insertion of commonly used documentation phrases and the use of the copy and paste command, which copies the content of one day’s notes into that of the next day’s notes, but at the same time, these tools have resulted in notes becoming increasingly bloated with sometimes outdated, irrelevant, and even erroneous information [1]. To trim down bloated clinical documentation, one approach of interest is to identify highly similar text snippets for the goal of removing such text. Wang et al [2,3] created the MedSTS data set—a clinical analogue of the natural language understanding benchmark task of assessing semantic textual similarity (STS)—to be a resource for this line of study. In this paper, we show the model, as well as subsequent improvements, that was used in the August 2019 National NLP Clinical Challenges (n2c2)/Open Health Natural Language Processing (OHNLP) Consortium semantic similarity shared task challenge [2], which featured the MedSTS data set.

In the broader natural language processing (NLP) community, STS assessment is a task in which the similarity of semantic meanings and content among natural language texts is calculated [3], and at the time of its release in late 2018, the bidirectional encoder representation from transformers (BERT) language model had the best published performance on the commonly used general English STS Benchmark (STS-B) data set [4]. For the MedSTS data set, it was shown that a BERT model that was fine-tuned to the biomedical domain also outperformed most prior state-of-the-art models [5]. The first iteration of the MedSTS challenge in 2018 (ie, prior to the release of BERT) saw 4 submissions involving the mixed use of traditional machine learning models, like random forests, and more recent deep learning architectures, like recurrent neural networks and convolutional neural networks. The 2019 MedSTS challenge saw over 30 submissions, and the majority of these submissions used BERT in some capacity. The increased number of submissions, as well as the increased average performance of submissions, can be attributed in large part to the recent progress in the development of language models, of which BERT is a popular example.

Despite such advances, researchers have noted that although language models demonstrate a small degree of commonsense reasoning and basic knowledge, such models are very limited in terms of their ability to generate factually correct text or even recall explicit facts from training data [6]. The attempts to mitigate these shortcomings of language models have often involved the use of graph representation learning techniques [7-9], which provide a natural way for working with knowledge in the form of graphs.

Recent progress in graph representation learning has given rise to 2 promising classes of methods that can be used in conjunction with NLP models to incorporate knowledge (either domain knowledge or commonsense knowledge)—graph convolutional networks (GCNs) [10] and knowledge graph embeddings (KGEs) [11].

GCNs generalize the notion of convolution from images to graph-structured data, thereby enabling the application of deep learning techniques on graphs. KGE methods are used to encode entities (nodes) and relationships (edges) in a knowledge graph into dense vector representations, much like word embeddings. KGEs provide a way of obtaining embeddings of concepts, and GCNs provide a natural way of using that information in the context of graph-based learning. For instance, GCNs can be used to initialize node features with pretrained KGEs.

In this study, we leveraged these recent advances in NLP and graph representation learning to develop a more knowledge-aware approach to assessing the MedSTS benchmark data set. We further investigated the benefits of other techniques, such as data augmentation, multisource ensembling, and knowledge distillation, and they resulted in competitive performance values for the 2019 n2c2/OHNLP Consortium semantic similarity shared task challenge.

## Methods

### Data Set

MedSTS is a data set of sentence pairs that were gathered from the clinical EHRs at Mayo Clinic. Deidentified sentences were selected based on their frequency of appearance and an assumption that frequently appearing sentences tend to contain less protected health information. Sentence pairings were arranged so that they had at least some degree of surface-level similarity. This was based on a combination of surface lexical similarity metrics. Broadly speaking, sentences generally fell into the following four categories: signs and symptoms, disorders, procedures, and medications. Further details are discussed in the original MedSTS paper [3]. For the 2019 n2c2/OHNLP competition and this study, a subset of annotated sentence pairs was examined; of the 2054 sentence pairs in this subset, 1652 (80.4%) were included in the training set, and 412 (20.1%) were included in the test set [2]. This subset was independently scored by 2 medical experts for semantic similarity. A 6-point (range: 0-5) rubric was provided to the annotators; 0 denotes complete dissimilarity, 1 indicates that 2 sentences are topically related but are otherwise not equivalent, and 5 represents complete similarity. The agreement between the two annotators received a weighted Cohen κ score of 0.67. The average of the two scores served as the gold standard against which STS systems would be evaluated [3].

### Concept Graph Construction

For each sentence in the MedSTS data set, we constructed a corresponding concept graph to represent the domain knowledge aspect of the data set. The concept graphs consisted of concepts that were tagged with a domain-specific tagger called *MetaMap* [12] and were mapped to a specified medical terminology. The idea was that such a graph would provide an additional representation of data containing explicit domain knowledge in the form of mapped concepts and their connections.

The Unified Medical Language System (UMLS) [13] is an important resource in biomedical and health care research that integrates many health and biomedical vocabularies and terminologies under a unified, interoperable system. MetaMap [12] is a widely used NLP tool that maps concepts in biomedical and clinical text to the UMLS Metathesaurus. We applied MetaMap on the MedSTS data set to extract biomedical and clinical entities that belonged to the Systematized Nomenclature of Medicine Clinical Terms (SNOMED CT) terminology of the UMLS. Thus, for each sentence, we obtained a corresponding list of extracted concepts, their concept unique identifiers, and semantic type information.

**Figure 1:**
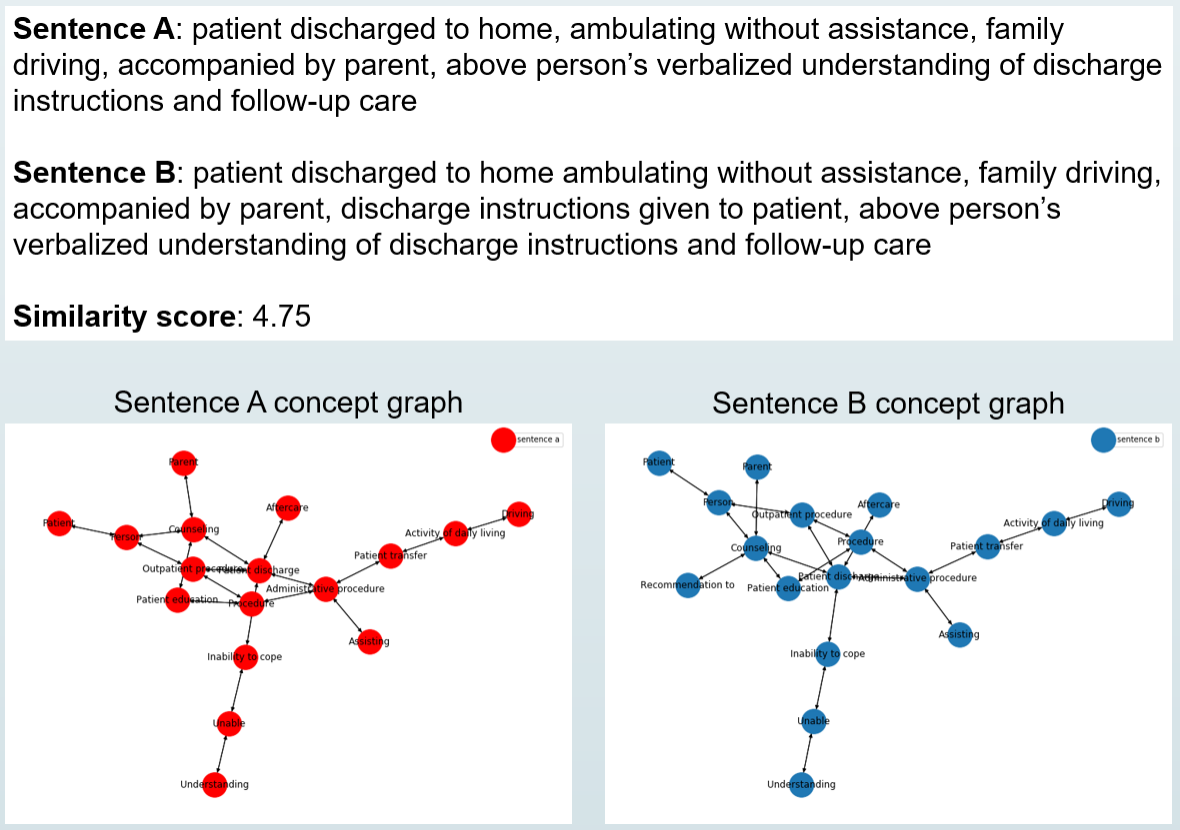
An example sentence pair, its similarity score, and a visualization of the corresponding concept graphs constructed from the concepts in the sentences.

We then constructed a graph of SNOMED CT terminology from the raw UMLS files by using the concepts (MRCONSO.RRF files) as nodes and by using the relationships (MRREL.RRF files) among them as edges. For simplicity, we only considered the connectivity information among the concepts and left the semantic information in the relation types for future work. Once we had a full SNOMED CT graph, we induced subgraphs for each sentence from MedSTS by taking the shortest paths between the concepts that were extracted from the sentences. More concretely, this was done by using the shortest path method via the Dijkstra algorithm in the Networkx [14] library. Although there are many possible ways of constructing such sentence graphs, we decided to use the simple and heuristic shortest path method to obtain a connected graph that represents each sentence. Examples of such concept graphs, along with their original sentences, are shown in (Figure 1).

### Data Augmentation

Given the small size of the data set, we decided to augment it by including additional domain knowledge from the MetaMap output files. Notably, there were 2 pieces of information that we chose to use—the preferred name of the mapped concept in the source terminology and the semantic type of the concept within the UMLS Semantic Network. The preferred name of a mapped concept can often be the same as how the concept appears in the text, but the preferred name sometimes provides potentially valuable information in the form of synonyms or abbreviation expansions. For example, in the text snippet “the patient was taken to the pacu in stable condition,” the term *pacu* is mapped to the UMLS concept *postoperative anesthesia care unit (PACU)*, thereby providing the full description of the abbreviated term. The strings of the preferred names of mapped concepts were simply appended to the original sentences in the data set. Likewise, the semantic types of the mapped concepts (eg, *Health Care Related Organization* for the term *pacu*) were appended to the original sentences. Another method we used was doubling the data set size by simply feeding the model a copy of the data set that included sentences formatted in the reverse order (ie, “sentence2:sentence1”). This yielded slightly better results than those that were obtained by simply doubling the number of training epochs, suggesting that feeding the model the reverse copy of the data set might have given it more explicit hints that the task was agnostic to the order of the sentences. Although the data augmentation techniques we used were simple and yielded moderate improvements in performance, a recent paper [15] provides more interesting approaches to data augmentation. In the paper [15], the authors used back-translation and performed segment reordering to augment the MedSTS data set.

### The BERT Model

The BERT model is a widely used NLP model that is part of the recently emerging class of language models that use transformers [16] as the building blocks. The BERT model stacks multiple layers of transformer-based modules that primarily use the multiheaded self-attention mechanism to encode text into dense embeddings. The model is trained by using the masked language modeling objective and the next sentence prediction objective, and pretrained models for BERT (and other similar models) are readily available on the HuggingFace Transformers library [17]. Shortly after the BERT model dominated the general NLP field, several variations of the BERT model that were adapted to the biomedical and clinical domains also became available [5,18,19]. These domain-adapted versions of the BERT model were trained on some combination of the Medical Information Mart for Intensive Care version 3 [20], PubMed [21], and PubMed Central [22] databases, and these versions have been shown to outperform the original BERT model in several clinical NLP tasks, suggesting that they are more appropriate for working with clinical text data sets like MedSTS.

### The GCN Method

Kipf et al [10] contributed to the popularization of graph neural networks by providing an efficient implementation method for GCNs and demonstrating their effectiveness in analyzing several benchmark graph data sets for graph classification, node classification, and link prediction. Variants of GCNs were soon applied successfully to various domains and problems, including the modeling of interactions in physical systems [23], drug-drug interactions [24], and text classification [25]. GCNs have become a popular deep learning model for working with graph-structured data, and we used GCNs to encode the concept graphs.

### KGE Methods

KGEs are a relatively novel class of methods for learning dense vector representations of entities and relations in multi-relational, heterogeneous knowledge graphs. Essentially, a KGE model maps entities and relations to embedding spaces by using a predefined scoring function. Due to their growing popularity and the availability of implementation methods, KGEs have recently been applied to various domains, including biomedical knowledge graphs [26]. Chang et al [26] showed that using KGEs for learning concept embeddings from medical terminologies and knowledge graphs is arguably a more principled and effective approach than using previous methods based on skip-gram–based models like Cui2Vec [27] or network embedding–based models like Snomed2Vec [28]. Although we initially used Cui2Vec for our entity vectors at the time of submission, we later used SNOMED CT KGEs after they became available in recent months.

### Augmenting BERT With KGEs for MedSTS

**Figure 2:**
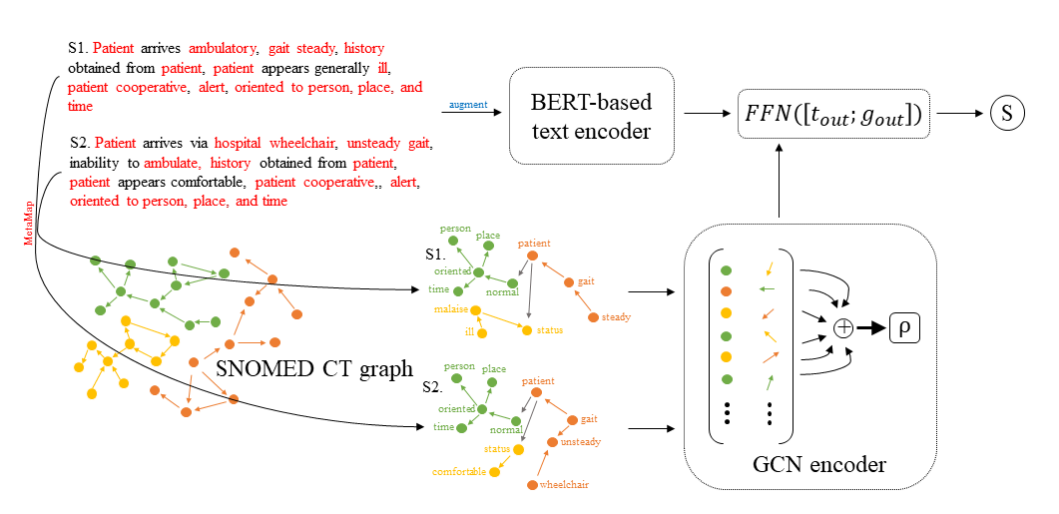
A simplified diagram of our pipeline. We passed the sentences through MetaMap to extract concepts belonging to the SNOMED CT and induced concept graphs by using the relationships among the terminology. We then passed the augmented sentence pairs to the text encoder and passed the concept graphs to the graph encoder. The outputs from the encoders were concatenated and passed to a fully connected layer to obtain an S. BERT: bidirectional encoder representation from transformers; FFN: feed-forward network; GCN: graph convolutional network; S: similarity score; S1: sentence 1; S2: sentence 2; SNOMED CT: Systematized Nomenclature of Medicine Clinical Terms.

We combined the components of GCNs and KGEs into a single model in the following way: we used a BERT-based model as our text encoder for the sentence pairs in MedSTS, used a GCN-based model as our graph encoder for the concept graphs that corresponded to the sentence pairs, initialized the node embeddings in the graphs by using pretrained SNOMED CT KGEs, concatenated the outputs of the text and graph encoders, and passed the final concatenated vector to a fully connected layer to obtain a semantic similarity score. We also tested the benefits of using the SNOMED CT KGEs by comparing this method to random initialization and initialization with Cui2Vec embeddings. A visualization of the pipeline is shown in Figure 2.

### Ensemble and Knowledge Distillation

After training our model, we took an ensemble to further improve the model’s performance. In accordance with Xu et al [29], we performed multisource ensembling with the following variants of BERT: BERT_base [4], SciBERT [30], ClinicalBERT [18], multi-task deep neural networks (MT-DNNs) [31], and BlueBERT [32]. Afterward, we performed knowledge distillation—an effective model compression method in which a smaller model is trained to mimic a larger model (ie, the ensemble). We used the predictions of the multisource ensemble model as soft labels in a teacher bounded regression loss function, in accordance with Chen et al [33], to train more individual models and obtain a final ensemble of the knowledge-distilled models.

## Results

We split the provided training set of MedSTS into 1313 training examples and 329 validation examples and reported the Pearson correlation coefficient for the held-out test set of 412 examples. The Pearson correlation coefficient was the chosen metric for the competition. We used the HuggingFace Transformers library for implementations related to language models, and we used PyTorch Geometric [34] for implementations of GCNs. Many of the default training and fine-tuning hyperparameters were used, while the following hyperparameters were tuned on the validation set: a learning rate of 1e^−4^ for BERT-based models (chosen from 5e^−5^, 1e^−4^, and 5e^−4^), a learning rate of 1e^−3^ for GCNs (chosen from 1e^−2^, 1e^−3^, and 1e^−4^), and 4 epochs (chosen from 3, 4, and 5 epochs).

Table 1 shows the contributions of the different components in the pipeline. Simply using the off-the-shelf BERT_base model and fine-tuning it on MedSTS yielded higher performance values compared to those of the 2018 submissions. Using ClinicalBERT and using our previously described data augmentation technique each yielded moderate gains.

**Table 1:** The results for the base model and each model version (an additional component was added to each system). The columns under Pearson correlation analysis show the scores for the test set (all) and the four subsets of the test set, which included sentences regarding patients’ conditions or statuses (status), patients’ education or interactions (education), patients’ medications (meds), and miscellaneous topics (miscellaneous).

Model name	Pearson correlation analysis				
	All, *r*	Status, *r*	Education, *r*	Meds, *r*	Miscellaneous, *r*
BERT_base	0.842	0.643	0.721	0.522	0.414
ClinicalBERT	0.848	0.662	0.735	0.541	0.425
ClinicalBERT-DA^a^	0.855	0.671	0.737	0.553	0.432
ClinicalBERT-DA + GCN_rand	0.861	0.675	0.742	0.532	0.427
ClinicalBERT-DA + GCN_cui2vec	0.863	0.682	0.753	0.536	0.442
ClinicalBERT-DA + GCN_snomedkge	0.868	0.693	0.761	0.562	0.463

^a^The ClinicalBERT-DA model refers to the ClinicalBERT model after data augmentation.

Adding a graph encoder, in addition to our other modifications, to incorporate the concept graphs resulted in minor improvements when the node embeddings were either initialized randomly or initialized with pretrained Cui2Vec embeddings. However, using SNOMED CT KGEs as the node features in the GCN resulted in an increase in performance, that is, an increase of 1.3% above the performance of ClinicalBERT (ie, after data augmentation), suggesting that SNOMED CT KGEs served as better starting representations of the concepts. It is worth noting that since the BERT-based text encoder is initialized with a pretrained checkpoint, it might be especially important to initialize the graph encoder with decent pretrained embeddings to allow the graph encoder to “catch up” with the text encoder. We called this best performing setting *ClinicalBERT_all*.

We also manually categorized the sentence pairs into the following four categories: sentences related to patients’ conditions and statuses (status), education or interactions with patients (education), medications (meds), and miscellaneous or clearly dissimilar topics (miscellaneous). The columns in Table 1 (those under *Pearson correlation analysis*) show the scores for the test set (all) and for the four categories described. Sentence pairs in the status and education categories received relatively higher scores, as expected, since many of the sentences and text snippets in these categories often repeated. Specifically, text snippets beginning with “patient arrives...,” “discussed the risks...,” or “identified illness as a learning need...” recurred noticeably in these two categories. Further, the medication and miscellaneous categories received relatively low correlation scores. For the miscellaneous category, this was expected, since many of the sentence pairs in this category were more difficult for the model to learn due to their greater variability. For the medication category, the gold-standard scores assigned by the annotators proved to be rather inconsistent and challenging to predict, even upon manual review by a medical expert.

Table 2 shows the results for ensembling and knowledge distillation. First, we took the ensemble of 10 ClinicalBERT_all models with slightly varied hyperparameters and saw a moderate increase in performance, as expected of ensembles. Second, in accordance with Xu et al [29], we took an ensemble of 10 models consisting of a variety of model types (BERT_base, SciBERT, ClinicalBERT, MT-DNNs, and BlueBERT), along with the graph encoder, based on their validation performance and saw a slight improvement. Finally, by using a teacher bounded regression loss function [33], we used the outputs of the multisource ensemble model as soft labels to train more best-setting models of different types and took an ensemble consisting of 10 such knowledge-distilled models for slight performance gain.

**Table 2:** Results for the ensembling of the best performing models from (ClinicalBERT_all), the ensembling of multiple language models (LMs; each with a graph convolutional network), and the ensembling of knowledge-distilled (KD) multisource ensembles.

Ensemble type	Performance, %
Ensemble of ClinicalBERT_all	87.5
Ensemble with multiple LMs	87.8
Ensemble of KD models	88.2
IBM-N2C2^a^	90.1

^a^The best performing model from the IBM team at the time of the competition was included for reference.

## Discussion

### Main Findings

We implemented a list of techniques in our pipeline for the clinical MedSTS benchmark task and reported slight to moderate improvements in performance for each technique. Using a pretrained, off-the-shelf, BERT-based model and fine-tuning it alone served as a strong baseline that outperformed all pre-BERT systems in the task. We found that our data augmentation technique helped slightly, but again, Wang et al [15] has provided more interesting and effective data augmentation approaches for MedSTS.

Adding a graph encoder to incorporate concept graphs into the pipeline yielded decent gains, especially when the graph encoder was initialized by using pretrained SNOMED CT KGEs. We stress that since the graph encoder was trained jointly with a pretrained text encoder, it is important to consider providing the graph encoder with pretrained embeddings as well, so that it does not fall too far behind in training.

As expected, ensembling leads to improved performance. Further improvements can be achieved by using language models from different sources as well as by performing knowledge distillation, which can be followed by the ensembling of the distilled models.

We also attempted to use several other techniques that did not yield any performance gains. First, we tried multi-task learning by using different general and clinical domain NLP data sets, including the Medical Natural Language Inference [35], Recognizing Question Entailment [36], and English STS-B [37] data sets, following an implementation of multi-task learning for MT-DNNs, but this approach did not result in any improvements and substantially increased the training time. Second, we tried manually annotating the MedSTS data for different sentence categories (medication, status, education, and miscellaneous). This was done as an auxiliary classification task (also an example of multi-task learning), but this did not lead to noticeable gains in performance. Lastly, we tried experimenting with different variants of GCNs, but we found that training multiple types of graph neural networks jointly with a large language model was difficult in terms of hyperparameter tuning and decided to limit our analysis to basic GCNs.

### Limitations of the Method

Although the results show that the strategies for data augmentation and the incorporation of domain knowledge through concept embeddings and GCNs do confer some benefit, we address some of the limitations in this section.

The data augmentation techniques we used involved including additional textual and semantic information from the MetaMap output and reversing the sentence order to double the data set size. There are many other potential data augmentation techniques in the general NLP field that could be useful. Notably, Wang et al [15] recently performed segment reordering and back-translation to substantially improve their model’s performance on a task.

As for the pretrained concept embeddings and GCNs, combining them with a large pretrained language model is still largely experimental. This can be improved by using recent developments in the field of graph representation learning, such as graph attention networks [38] and graph matching networks [39].

### Limitations of the Data Set

Both the positive and negative findings should be considered with caution due to the abundance of the potential ways of implementing each component as well as the size and quality of the data set, which was relatively smaller and of lower quality compared to data sets in mainstream, nonclinical NLP domains that have less complicated access to labeled data.

After working closely with the data set for several months, we noticed that certain sentence pairs had large irregularities in terms of their scores from the two annotators of the data set. This was the most notable in the sentence pairs that discussed medications; often, these sentence pairs described the prescribing of medications to patients and differed in terms of dosing or drug class. At one level of categorization, the similarity of a sentence pair related to prescribing could be seen as high, regardless of the medication class or dosing. At another level of categorization, it appeared that several such pairs were noted to be of low similarity when the medications or dosing regimens differed. This discrepancy in scoring also seemed to differ depending on the drug classes being mentioned. Without knowing which annotator was responsible for a given score, it is difficult to speak conclusively, but we speculate that certain drug classes were of greater salience to each annotator. As an example, someone with a specialty in a mental health may subjectively perceive 2 different psychiatric medications of different classes to be quite different but view cardiology drugs to be subjectively more similar. In contrast, an individual in the field of cardiology may perceive various cardiology drugs as being different but may perceive drugs in the psychiatric medications category overall as being more similar. Such differences in perspectives may also be influenced by aspects of an annotator’s practice, such as whether their practice occurs in inpatient settings, outpatient settings, the operating room, or the medical clinic.

Many of the scoring irregularities may have been related to the nature of the task of rating subjective similarity. One approach to mitigating annotator bias, as discussed in the original MedSTS paper [3], is to increase the number of annotators and set the average score as the gold standard. For example, in the English STS-B, 5 annotators were used for each sentence, and annotators were limited to a certain number of sentence pairs that they could annotate [37]. Although such an approach can be prohibitively expensive due to the need to hire enough medical annotators and be very cumbersome to implement for clinical text due to patient privacy protections, another approach for the case of having few annotators could be to reveal potentially biasing factors toward annotation, such as clinical background, or to assign an annotator ID to each score. Stating the biases or allowing teams to model the annotator biases may help with understanding scoring irregularities that may be difficult to resolve without the use of specifically tailored algorithm designs or features, which require specific domain knowledge to adapt to unique annotator biases.

Despite our concerns with the fundamental difficulty of objectively rating subjective semantic similarity, the high Pearson correlation coefficient achieved by our model suggests that the task is still largely tractable. MedSTS also remains one of the few, if not only, publicly available data sets for studying clinical STS in EHRs. We hope that our suggestions may introduce additional strategies for modeling the variance from subjective elements and provide some insights to future data set annotation processes for this important yet challenging problem.

### Conclusions

As participants of the 2019 n2c2/OHNLP shared task challenge, we developed a system for the clinical MedSTS benchmark task by combining BERT-based text encoders and GCN-based graph encoders in order to incorporate domain knowledge into the NLP pipeline. We also experimented with other techniques involving data augmentation, pretrained concept embeddings, ensembling, and knowledge distillation to further increase our model’s performance. Although our results lagged behind those of the top scoring model at the n2c2 workshop, the incorporation of domain knowledge into deep learning NLP models via graph-based methods was a new advance in clinical NLP. We highlight our concerns about the impact of specific difficulties with subjective semantic similarities in data set annotation, but overall, we believe that clinical semantic similarity remains an important topic of study, and continued work on the MedSTS benchmark—one of the few clinical STS data sets available—will yield advances in processing valuable unstructured data in EHRs. The MedSTS data set should continue to be improved and enlarged through the further careful annotation of the original pool of sentence pairs, and future work should explore novel methods that can effectively leverage both linguistic and domain knowledge.

## Abbreviations

BERT: bidirectional encoder representation from transformers
EHR: electronic health records
GCN: graph convolutional network
KGE: knowledge graph embedding
MT-DNN: multi-task deep neural network
n2c2: National NLP Clinical Challenges
NLP: natural language processing
OHNLP: Open Health Natural Language Processing Consortium
SNOMED CT: Systematized Nomenclature of Medicine Clinical Terms
STS: semantic textual similarity
STS-B: Semantic Textual Similarity Benchmark
UMLS: Unified Medical Language System
